# Arsenite as an Electron Donor for Anoxygenic Photosynthesis: Description of Three Strains of *Ectothiorhodospira* from Mono Lake, California and Big Soda Lake, Nevada

**DOI:** 10.3390/life7010001

**Published:** 2016-12-26

**Authors:** Shelley Hoeft McCann, Alison Boren, Jaime Hernandez-Maldonado, Brendon Stoneburner, Chad W. Saltikov, John F. Stolz, Ronald S. Oremland

**Affiliations:** 1 U.S. Geological Survey, Menlo Park, CA 94025, USA; 2 Department of Microbiology and Environmental Toxicology, University of California, Santa Cruz, CA 95064, USA; alisonboren@gmail.com (A.B.); jhernan4@ucsc.edu (J.H.-M.); bstonebu@ucsc.edu (B.S.); saltikov@ucsc.edu (C.W.S.); 3 Department of Biological Sciences, Duquesne University, Pittsburgh, PA 15282, USA; stolz@duq.edu

**Keywords:** arsenite, anoxygenic photosynthesis, soda lakes, *arxA*, *Ectothiorhodospira*

## Abstract

Three novel strains of photosynthetic bacteria from the family Ectothiorhodospiraceae were isolated from soda lakes of the Great Basin Desert, USA by employing arsenite (As(III)) as the sole electron donor in the enrichment/isolation process. Strain PHS-1 was previously isolated from a hot spring in Mono Lake, while strain MLW-1 was obtained from Mono Lake sediment, and strain BSL-9 was isolated from Big Soda Lake. Strains PHS-1, MLW-1, and BSL-9 were all capable of As(III)-dependent growth via anoxygenic photosynthesis and contained homologs of arxA, but displayed different phenotypes. Comparisons were made with three related species: *Ectothiorhodospira shaposhnikovii* DSM 2111, *Ectothiorhodospira shaposhnikovii* DSM 243^T^, and *Halorhodospira halophila* DSM 244. All three type cultures oxidized arsenite to arsenate but did not grow with As(III) as the sole electron donor. DNA–DNA hybridization indicated that strain PHS-1 belongs to the same species as *Ect. shaposhnikovii* DSM 2111 (81.1% sequence similarity), distinct from *Ect. shaposhnikovii* DSM 243^T^ (58.1% sequence similarity). These results suggest that the capacity for light-driven As(III) oxidation is a common phenomenon among purple photosynthetic bacteria in soda lakes. However, the use of As(III) as a sole electron donor to sustain growth via anoxygenic photosynthesis is confined to novel isolates that were screened for by this selective cultivation criterion.

## 1. Introduction

The genus *Ectothiorhodospira* of the family Ectothiorhodospiraceae is composed of photosynthetic purple sulfur bacteria dependent on saline and alkaline growth conditions [1]. Isolates of this genus occur in marine environments as well as in the more chemically-extreme regimen of hypersaline and alkaline (soda) lakes [1]. They typically grow via anoxygenic photosynthesis using reduced sulfur compounds, hydrogen, or organic compounds (e.g., acetate, malate) as electron donors [2]. More recently, arsenite (As(III)) was also found to serve as an electron donor for anoxygenic photosynthesis in Mono Lake biomes: both in an *Ectothiorhodospira*-dominated enrichment culture cultivated from Mono Lake sediments [3] and in red biofilms located in the lake’s hot springs [4]. *Ectothiorhodospira* strain PHS-1 was isolated from these biofilms and was shown to grow as a photoautotroph, using As(III) as its sole electron donor [4].

Both Mono Lake and Big Soda Lake waters contain abundant dissolved inorganic arsenic, 200 and 20 µM, respectively [5]. Mono Lake has been the more intensively studied of the two with respect to arsenotrophy [6] and has been found to support a breadth of arsenotrophic microbes. Arsenate-respiring bacteria in Mono Lake mediate the oxidation of organic matter [7] as well as the oxidation of inorganic electron donors like sulfide [8]. Diverse chemoautotrophic bacteria can oxidize arsenite (As(III)) to arsenate (As(V)) under aerobic or and anaerobic conditions, depending upon the species. The enzymes that support these pathways include a respiratory As(V) reductase (ArrAB-ArrABC) and an As(III) oxidase (AioBA; formerly referred to as AroAB, AoxAB or AsoAB). The arsenate reductase, ArrA, and arsenite oxidase, AioA, are members of the DMSO reductase family of molybdenum containing oxidoreductases and function catalytically to reduce arsenate or oxidize arsenite, respectively. ArrB and AioB are FeS subunits that participate in electron transfer to/from ArrA/AioA, respectively. ArrC is predicted to function as a quinol-oxidizing, membrane-anchoring subunit for ArrAB. *Alkalilimnicola ehrlichii* strain MLHE-1, isolated from Mono Lake water, grows chemo-autotrophically by coupling the oxidation of As(III) with the reduction of nitrate [9,10]. It lacks an arsenite oxidase (i.e., AioBA) and instead uses a different arsenite oxidase enzyme, ArxA (encoded by *arxB_1_AB_2_C*), which more closely resembles ArrA than AioA, for oxidizing As(III) to As(V) [11]. ArxB and ArxC are predicted to function as FeS- and membrane-anchoring/quinone-reducing subunits, respectively. The *arxA* gene is aligned more closely with *arrA* yet is comparatively distant from *aioA*, implying an evolutionary relationship with the former but not the latter [12]. *Ectothiorhodospira* strain PHS-1 also lacks the *aio* gene cluster but contains *arx* genes (including *arxA*) in a similar arrangement as MLHE-1 [13,14]. *arxA* genes were also found in the biofilms of the hot spring pools on Paoha Island [13]. Subsequent studies identified *arxA*-like sequences in Mono Lake, freshwater Hot Creek sediments, within the annotated metagenomes of several hot springs in Yellowstone National Park [14], and from sediments collected from Tukh Lake, an arsenic-rich soda lake located in Mongolia [15], indicating that *arxA*-type arsenite oxidases may be widespread in nature. Here we report the further characterization of strain PHS-1 and the isolation of two new strains of *Ectothiorhodospira*: strain MLW-1, isolated from shoreline sediments of Mono Lake and strain BSL-9, isolated from Big Soda Lake marsh water, all of which grow using As(III) as the sole electron donor that supports anoxygenic photosynthesis. In contrast, although closely related photosynthetic cultures from type collections could oxidize As(III), they were unable to sustain As(III)-dependent growth. Our results suggest that arsenotrophic growth among photosynthetic bacteria is more widespread than is currently recognized.

## 2. Materials and Methods

### 2.1. Isolation and Growth

Strain PHS-1 was isolated from red pigmented biofilms growing on rock surfaces in a hot pool (~45 °C) located on Paoha Island in Mono Lake, CA (37°59′ N; 119°01′ W ) and was maintained in an anaerobic liquid basal salts medium with As(III) as the electron donor, as previously described [4]. Strain MLW-1 was enriched from sediment collected from the littoral zone on the north shore of Mono Lake (38°04′ N; 118°59′ W). The enrichment was maintained in an artificial medium containing the following (g/L): NaCl (60), (NH_4_)_2_SO_4_ (0.1), KH_2_PO_4_ (0.08), K_2_HPO_4_ (0.15), MgSO_4_·7H_2_O (0.025), Na_2_CO_3_ (10.6), NaHCO_3_ (4.2), Na_2_WO_4_ (0.00001), and Widdel et al.’s [16] trace elements solution (5 mL). The pH was adjusted to 9.8, bubbled with O_2_-free N_2_ for 30 min and 9 mL of medium was dispensed into anoxic, N_2_-flushed Balch-type tubes (~25 mL), which were then crimp sealed and sterilized by autoclaving (121 °C, 250 kPa for 60 min). The medium was amended with 2 mM As(III), 0.1 mM sulfide and 10 mL/L of a vitamin solution [17], all from sterile anaerobic stock solutions and incubated in the light (25 watt bulb; 1000 lux) at 28 °C. After several transfers in liquid medium, the enrichment culture was streaked onto 1.5% agar medium contained in agar bottle plates [18], sealed under N_2_, and incubated in the light at 28 °C. All manipulations were done using sterile technique in an anaerobic glove box. Pink colonies were noted after about 7–14 days and were picked and re-inoculated into the original sterile liquid medium. The purity of the culture was determined by morphological uniformity and by no ambiguous 16S rRNA sequences. MLW-1 was maintained with 2 mM As(III) as the electron donor, 0.1 mM sulfide as a reducing agent and incubated at 28 °C statically in the light.

Strain BSL-9 was isolated in 2011 from water collected in the shallow swamp region of Big Soda Lake (39°31’ N, 118°53’ W) and the enrichment was performed as described for strain MLW-1 using the following artificial medium: (g/L^−1^): NaCl (25.0), KH_2_PO_4_ (0.24), K_2_HPO_4_ (0.3) (NH_4_)_2_SO_4_ (0.23), MgSO_4_ (0.12), Na_2_CO_3_ (5.3), NaHCO_3_ (2.1), yeast extract (0.02), vitamin B12 (0.0002), SL10 trace element solution (1 mL) [19], vitamin mix (10 mL) [20], and adjusted to pH 9.7. Once established, BSL-9 was typically grown in the light (~2000 lux) at 30 °C with 2 mM As(III) as the electron donor.

*Ectothiorhodospira shaposhnikovii* (DSM 2111) and *Ectothiorhodospira shaposhnikovii* (DSM 243^T^) were maintained in ATCC medium #1448 at pH 9 with 10 mM acetate and 2 mM sulfide as electron donors. *Halorhodospira halophila* (DSM 244) was maintained in DSM medium #253 at pH 8.5 with 6 mM succinate and 4 mM sulfide as electron donors. All three of these strains were incubated in the light (25 W bulb, ~1000 lux) at 28 °C.

Growth of cultures was monitored by optical density at 680 nm (600 nm for strain BSL-9) by spectrophotometry. All growth experiments were conducted in 25-mL Balch tubes filled with 9 mL of anoxic medium and N_2_ headspace and inoculated with 1 mL of liquid culture. Tubes were illuminated by a 25 W tungsten light bulb and incubated statically at 28 °C. Growth of strain MLW-1 was tested with 2 mM As(III) (initial concentration) as the electron donor and 0.1 mM sulfide as a reducing agent. During incubation, arsenic speciation was monitored and, as As(III) was removed, it was replaced with further injections of 2 mM As(III) so as to avoid the overt growth-preventing toxicity of initially adding too much arsenite (e.g., 5–10 mM) [4]. Strain BSL-9 was grown in BSM with an initial arsenite concentration of 0.5 mM at approximately 2000 lux. Growth was determined from the absorbance at 600 nm. As growth increased, several subsequent ~0.5 mM arsenite additions were made to cultures as is outlined above for strain MLW-1.

To examine whether PHS-1 and MLW-1 could use electron donors other than As(III) for growth, transfers of 1.0 mL inoculum were made into crimp-sealed Balch tubes containing 9 mL of medium. Electron donors were added from sterile anoxic stock solutions to yield the final concentrations given in Table 1, while controls lacked electron donors. Growth was monitored by optical density at 680 nm. If growth occurred, the culture was transferred twice sequentially into the same medium for confirmation of sustained growth. Due to PHS-1’s very high 16S rRNA sequence similarity (>97%) to *Ect. shaposhnikovii* DSM 2111 and *Ect. shaposhnikovii* DSM 243^T^, these organisms were tested alongside strain PHS-1 and MLW-1. *Ect. shaposhnikovii* DSM 2111 was tested for growth on all electron donors listed in Table 1, while *Ect. shaposhnikovii* DSM 243 was tested only with the following electron donors: lactate, acetate, and butyrate. For strain BSL-9, a liquid culture was first grown in BSM with 10 mM malate (pH 9.7) for three days and diluted 1/10 in blank medium. The diluted culture was inoculated (300 µL) into 10 mL of anaerobic medium containing the following organic acids as electron donors (10 mM): malate, acetate, propionate, pyruvate, succinate, fumarate, and lactate, followed by incubation at 30 °C. Growth (OD_600 nm_) was monitored daily. No growth was observed in the absence of any added organic acid substrates.

The ability of the three strains to grow over a range of salinity, pH and temperature was examined by growing the organisms with 10 mM acetate in the light. Salinities were varied by altering the concentration of NaCl in the media: (0–175 g/L; strain MLW-1; 0–80 g/L strain PHS-1; 0–100 g/L strain BSL-9. The pH range was tested using the media described above, buffered with Na_2_CO_3_/NaHCO_3_ for pH range 8.5–12 and adjusted with concentrated HCl or 6N NaOH. For pH range 6.5–8.5, the buffer was HEPES/ Na_2_CO_3_ (PHS-1: 35.7 g/L HEPES + 5.3 g/L Na_2_CO_3_; MLW-1: 23.8 g/L HEPES + 10.6 g/L Na_2_CO_3_). Specific growth rates (μ) were determined by a least-squares linear fit to the following formula: μ = ln (N/N_0_)/t, where N_0_ and N are OD_680nm_ values for the time interval displaying exponential growth.

### 2.2. Sequencing and Phylogenetic Analysis

The 16S rRNA gene sequencing for PHS-1 was performed as described in Kulp et al. [4]. Genomic DNA of strains MLW-1 and BSL-9 were extracted using the Qiagen DNeasy Blood and Tissue Kit (Qiagen, Hilden, Germany). The following PCR conditions were used to amplify the 16S rRNA gene: 25 µL 2× Taq mixture (Promega), 5 µL 10× reaction buffer, and 5 µL each of 2 µM forward and reverse primer (8F, 5’-GAGTTTGATCCTGGCTCAG-3’ and 1492R, 5’-GGTTACCTTGTTACGACTT-3’). The PCR products were cloned using the Invitrogen TA-TOPO cloning kit according to the manufacturer’s instructions. Clones were screened for correctly sized inserts (~1500 bp). Sequencing was done on replicate clones by Sequetech DNA Sequencing Service (Mountain View, CA, USA) with forward (M13F) and reverse (M13R) vector-specific primer. For each organism, the forward and reverse sequencing reads were manually inspected and assembled into a contiguous sequence based on the overlapping regions resulting in a nearly complete 16S rRNA gene.

Taxa inferences were done using NCBI BLAST and the partial 16S rRNA genes for MLW-1, BSL-9, and PHS-1. Additional 16S rRNA gene sequences of closely related *Ectothiorhodospira* strains were obtained from NCBI. The sequences were aligned using CLUSTAL X and a neighbor-joining tree was constructed using PAUP* 4.0b [22].

DNA-DNA hybridization assays of strain PHS-1 against *Ect. shaposhnikovii* DSM 2111 and *Ect. shaposhnikovii* DSM 243^T^ were conducted at the Deutsche Sammlung von Mikrooganismen und Zellkulturen (DSMZ). Cells were disrupted by using a Constant Systems TS 0.75 KW (IUL Instruments, Königswinter, Germany) and the DNA in the crude lysate was purified by chromatography on hydroxyapatite as described by Cashion et al. [23]. DNA-DNA hybridization was carried out as described by De Ley et al. [24] under consideration of the modifications described by Huss et al. [25] using a model Cary 100 Bio UV/VIS-spectrophotometer (Agilent, Santa Clara, CA, USA) equipped with a Peltier-thermostatted 6 × 6 multi-cell changer and a temperature controller with in situ temperature probe (Varian). The G + C base composition of strain MLW-1 was determined by HPLC at the DSMZ.

### 2.3. Detection of arxA and aioA

Each strain was screened by PCR for *arxA* using primers BSL9arxA295f (5’-GGCGCCTATTTCCTGTATGA-3’) and BSL9arxA912r (5’-GGCAAAGTCACCCACAAACT-3’). The arsenite oxidase gene, *aioA* was detected using primers reported in Sultana, et al. [26]: AOX-F-A2: 5’-TGCATCGTCGGCTGYGGNTAY-3’ and AOX-R-E2: 5’-TTCGGAGTTATAGGCCGGNCKRTTRTG-3’. The *arxA* PCR conditions were: 200 nM of each primer, 2× Taq mixture from Promega, and template DNA (~10–50 ng). The following thermocycle profile was used: an initial denaturation at 95 °C for 5 min, followed by 30 cycles of 95 °C for 30 s, 55 °C primer annealing for 30 s, 72 °C extension for 1 min, followed by a final extension at 72 °C for 5 min. The *aioA* (previously referred to as *aoxB*) PCR conditions were similar to *arxA* however a 57 °C primer annealing temperature was used. The presence of 550 bp or 670 bp bands on an agarose gel was indicative of successful amplification of *arxA* and *aioA*, respectively. The PCR products were cloned using the Invitrogen TA-TOPO cloning kit and sequenced using the Sanger method. BLAST analysis of the partial *arxA* and *aioA* sequences were done using NCBI databases confirming the initial identity of the amplified arsenite oxidase sequences. The PHS-1 genome sequence is reported in Zargar et al. [14] and is available through NCBI accession number PRJNA68693. The BSL-9 genome sequence was completed using PacBio sequencing service at UC Davis, CA. High molecular weight DNA was prepared from a freshly grown culture initiated from a single colony that was streak plated on an agar medium containing acetate and arsenite. DNA was extracted using the Qiagen Genomic Tip 500-G (Qiagen, Germantown, MD, USA) according to the manufacturer’s instructions. The genome assembly and annotation are described in Hernandez-Maldonado et al. [27] and available through the NCBI BioProject and BioSample accession numbers PRJNA232800 and SAMN03795182, respectively.

### 2.4. Electron Microscopy

Samples for scanning electron microscopy (SEM) were prepared according to Smith et al. [28]. For transmission electron microscopy, cells were fixed by addition of glutaraldehyde directly to the medium (liquid cultures) for a final concentration of 2.5%. Samples were post-fixed with 1% osmium tetroxide in 0.5 M sodium acetate, *en bloc* stained with uranyl acetate (1% in deionized water), dehydrated in an ethanol series and propylene oxide, followed by infiltration with Spurr’s embedding medium as described in Switzer Blum et al. [29]. Samples were observed on a JEOL 100CX or 1210 transmission electron microscope (JEOL USA, Peabody, MA, USA) at 60 kV. Digital images were minimally processed for brightness and contrast.

### 2.5. Analytical Methods

Arsenic speciation and concentration and succinate concentration were determined by high-performance liquid chromatography (HPLC) using a Thermo Scientific Ultimate 3000 chromatograph (Thermofisher, Sunnyvale, CA, USA) with a UV-VIS detector (RS variable wavelength detector) set at 190 nm. Arsenate and arsenite were separated using two columns in series (Bio-Rad Aminex HPX-87H and Hamilton PRP X300) with a 0.016 N H_2_SO_4_ eluent at a flow rate of 0.6 mL/min. Retention times for arsenate and arsenite were 11.9 and 16.6 min, respectively. The retention time for succinate was 32 min. Sulfide was determined spectrophotometrically [30].

## 3. Results

### 3.1. Growth Experiments

**Figure 1:**
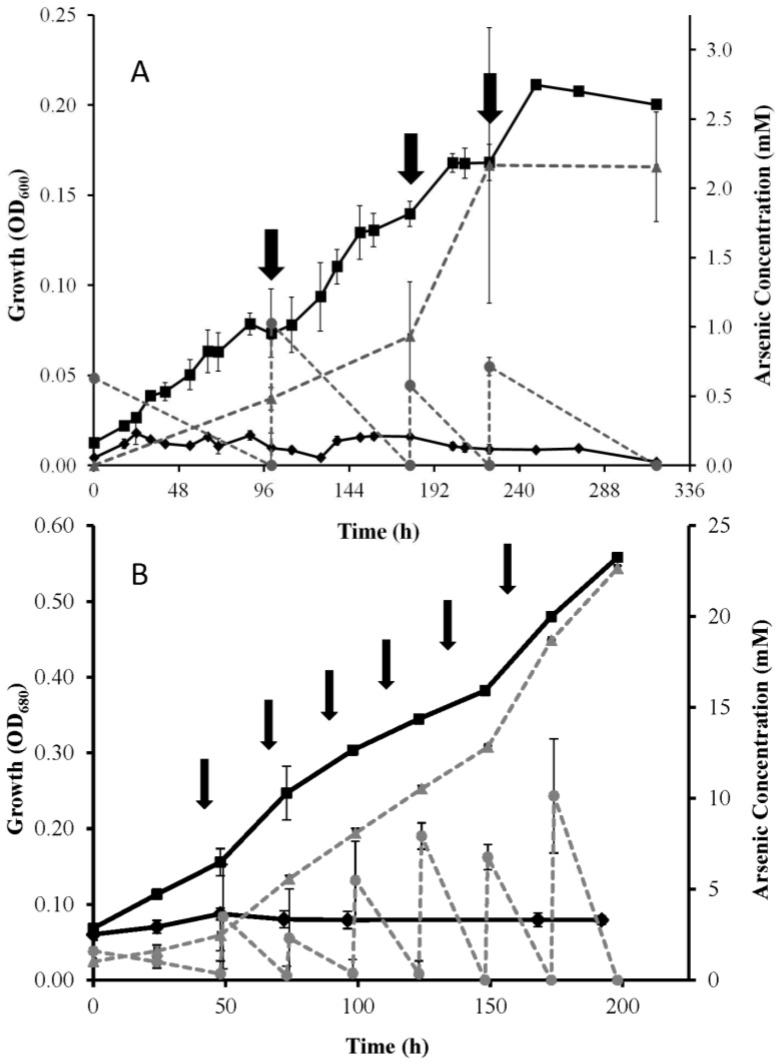
Anaerobic growth of BSL-9 (**A**) and MLW-1 (**B**) grown in the light with As(III) (■), or without added electron donor (♦). Arsenic speciation: As(V), (▲); As(III), (●). Symbols represent the means for three separate cultures, and error bars indicate ± standard deviation. Arrows indicate when additional As(III) was added to the cultures.

*Ectothiorhodospira* strain PHS-1 was previously shown to achieve light-dependent anaerobic growth by oxidizing As(III) to As(V). A series of pulsed additions of ~1–2 mM As(III) was made over time so as to avoid an initial inhibitory effect of this toxicant when applied at higher concentrations [4]. Similarly, strains MLW-1 and BSL-9 both grew as photoautotrophs by oxidizing pulsed additions of As(III) to As(V) under anaerobic conditions (Figure 1A,B). Growth of both BSL-9 and MLW-1 did not occur without As(III) (Figure 1A,B).

**Figure 2:**
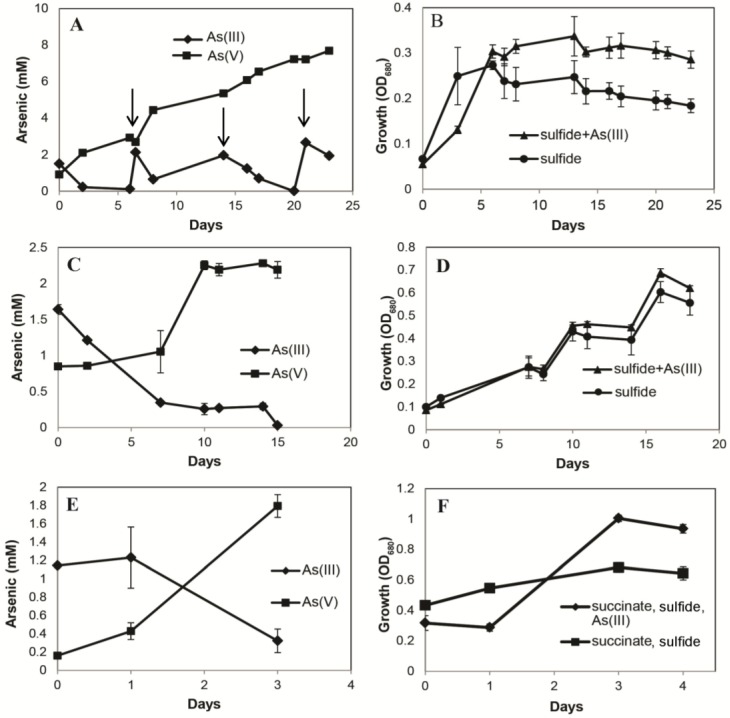
Phototrophic arsenite oxidation (left column) and growth (right column) in three closely related photosynthetic bacteria from culture collections. (**A**) As(III) oxidation to As(V) by *Ectothiorhodospira shaposhnikovii* DMS 243^T^ with added sulfide (2 mM). Arrows indicate additions of 2 mM As(III); (**B**) Growth of *Ect. shaposhnikovii* DSM 243^T^ in the light with As(III) + sulfide and sulfide alone; (**C**) As(III) oxidation to As(V) by *Ectothiorhodospira* *shaposhnikovii* DSM 2111 with added sulfide (2 mM); (**D**) Growth of *Ect. shaposhnikovii* DSM 2111 in the light with As(III) + sulfide and with sulfide alone; (**E**) As(III) oxidation to As(V) by *Halorhodospira halophila* with added sulfide (2 mM) and succinate (6 mM); (**F**) Growth of *H. halophila* in the light with As(III) + succinate + sulfide compared to succinate + sulfide. Symbols represent the mean of three cultures, and bars indicate ± SD.

Three closely related species from culture collections were tested for their ability to grow by oxidizing As(III) in the light. *Ectothiorhodospira shaposhnikovii* (DSM 243^T^) oxidized As(III) to As(V) in the light under anaerobic conditions, but growth occurred only when abundant sulfide (2 mM) was present (Figure 2A) and growth was slightly greater in cultures given As(III) plus sulfide than in cultures grown only with 2 mM sulfide (Figure 2B). Growth did not occur in this strain when As(III) was provided as the sole electron donor (Figure S1). *Ectothiorhodospira shaposhnikovii* (DSM 2111) oxidized As(III) to As(V) in the light under anaerobic conditions when 2 mM sulfide was present (Figure 2C), but As(III) did not notably enhance growth (Figure 2D). As with *Ect. shaposhnikovii* DSM 243, growth did not occur when As(III) was provided as the sole electron donor (Figure S1). *Halorhodospira halophila* (DSM 244) oxidized As(III) to As(V) in the light under anaerobic conditions in the presence of succinate plus sulfide (6 mM and 0.5 mM, respectively)(Figure 2E). Growth of *H. halophila* was greater in the As(III) plus succinate/sulfide condition when compared with the succinate/sulfide that lacked As(III) (Figure 2F). Growth did not occur when As(III) was the sole electron donor (Figure S2).

Strains MLW-1, PHS-1, and BSL-9 were tested for photosynthetic growth on a variety of other electron donors besides As(III). Strains MLW-1 and PHS-1 were able to use sulfide, thiosulfate, and sulfur, but not hydrogen, while strain BSL-9 grew on sulfide, thiosulfate, and sulfur (hydrogen was not tested). Photo-heterotrophic growth was observed in MLW-1 and PHS-1 with acetate, lactate, propionate, succinate, malate, and pyruvate but not with butyrate. BSL-9 also grew on these organic acids in addition to fumarate. Strain MLW-1 also grew with glucose and fructose, while strain PHS-1 did not (Table 1).

**Figure 3:**
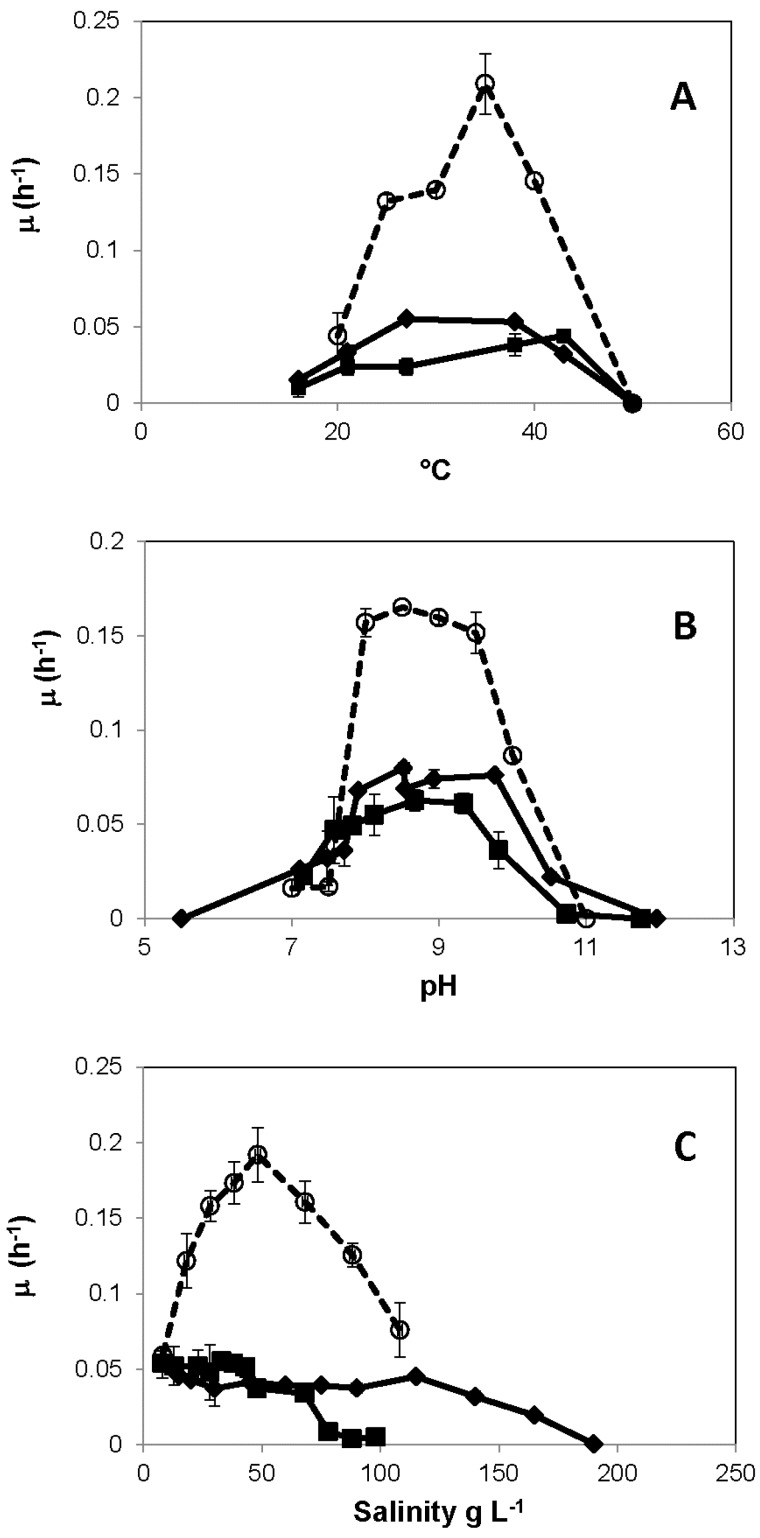
Optimal growth parameters for (■) strain PHS-1, (♦) strain MLW-1 and (○) strain BSL-9: (**A**) temperature; (**B**) pH; and (**C**) salinity. Symbols represent the mean of three cultures, and bars indicate ±SD.

Optimum growth of strains MLW-1, PHS-1, and BSL-9 (Figure 3) occurred at 27 °C, 43 °C, and 35 °C, respectively. The three strains were alkaliphilic. Strain MLW-1 exhibited a maximum growth rate in the pH range of 7.9–9.8, PHS-1 grew best at pH range 8.7–9.3, and BSL-9 grew best over a pH range of 8.0–9.5. Growth of strain MLW-1 occurred over a salinity range of 15–165 g·L^−1^, with the highest growth rates occurring at 15–115 g·L^−1^. Strain PHS-1 grew over a salinity range of 8–98 g·L^−1^, with the optimum growth rate at 33 g·L^−1^. BSL-9 grew optimally at a salinity of 48 g·L^−1^.

### 3.2. Morphological Characteristics of the Isolates

**Figure 4:**
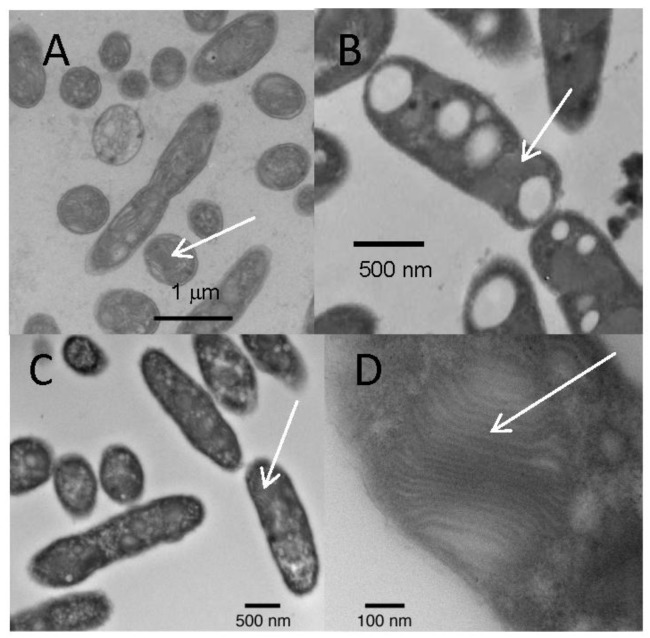
Thin sections of (**A**) strain PHS-1; (**B**) strain MLW-1; (**C**) strain BSL-9; and (**D**) strain BSL-9, lamellar intracytoplasmic membranes shown in detail. Arrows indicate locations of lamellar structures.

**Figure 5:**
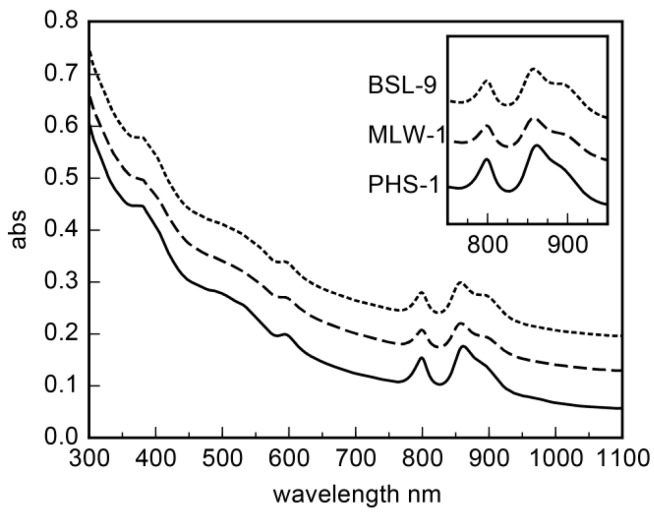
Absorption spectra of intact cells of strains BSL-9, MLW-1, and PHS-1, showing characteristic absorption peaks at 800 nm and 850 nm.

Strain PHS-1 is a gram-negative, motile rod (dimensions: 1.5–2.5 × 0.5–0.7 μm). Thin sections of PHS-1 cells displayed lamellar intracytoplasmic membranes (ICMs) (Figure 4A). Strain MLW-1 is a bean-shaped, gram-negative motile rod (dimensions: 1.0–2.2 × 0.5–0.75 μm). Thin sections of MLW-1 and BSL-9 cells revealed lamellar ICMs (Figure 4B–D). The cell suspensions of all three strains grown in the light were a deep red color. Absorption spectra of whole cells showed maxima at 800 and 850 nm, indicative of bacteriochlorophyll *a* (Figure 5).

### 3.3. Phylogenetic Analysis

**Figure 6:**
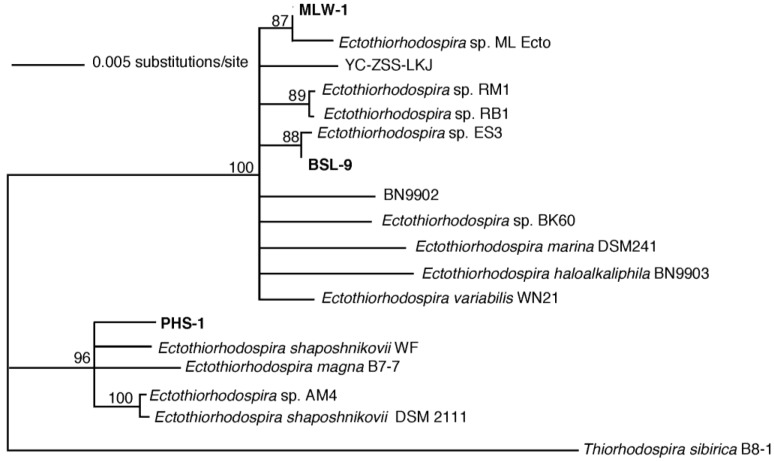
Phylogenetic tree base on 16S rRNA gene sequences for MLW-1, BSL-9 and PHS-1. BLAST searches using NCBI were done individually for MLW-1, BSL-9 and PHS-1 to find the closest phylogenetic relatives. Additional 16S rRNA gene sequences of closely related *Ectothiorhodospira* strains were obtained from NCBI. The sequences were aligned using CLUSTAL X and a neighbor joining tree was constructed using PAUP* 4.0b [22]. Accession numbers: *Ectothiorhodospira* “Borgoria Red” strain RM1 (AF384206), *Ectothiorhodospira* “Borgoria Red” strain RB1 (AF384207), YC-ZSS-LKJ151 (KP174461), *Ectothiorhodospira* sp. ML Ecto (EU341299), *Ectothiorhodospira* sp. strain ES3 (EU908046), BN9902 (X93475), *Ectothiorhodospira* sp. strain BK60 (KP681556), *Ectothiorhodospira marina* DSM 241 (NR044898), *Ectothiorhodospira haloalkaliphila* strain BN9903 (NR044900), *Ectothiorhodospira variabilis* strain WN21 (AM943125), *Ectothiorhodospira shaposhnikovii* strain WF (KJ586501), *Ectothiorhodospira magna* strain B7-7 (NR108987), *Ectothiorhodospira* sp. strain AM4 (EU252492), *Ectothiorhodospira shaposhnikovii* strain DSM 2111 (FR733667), *Thiorhodospira sibirica* strain B8-1 (HQ877088).

The results of the phylogenetic analysis indicated that strains PHS-1, MLW-1, and BSL-9 belong to the genus *Ectothiorhodospira* (Figure 6). BLAST analysis indicated the closest relative to MLW-1 was *Ectothiorhodospira* “Borgoria Red” strain RM1 (AF 384206) with a 99% sequence identity. The closest relative to BSL-9 was also strain RM1, but with a 98% sequence identity. The closest relative to PHS-1 was *Ectothiorhodospira shaposhnikovii* strain DSM 2111 with a 99% sequence identity. These results were based on percent coverage, as other strains had as high a percentage sequence identity. The phylogenetic tree revealed that *Ectothiorhodospira* sp. strain ML Ecto, also from Mono Lake, aligned with MLW-1. The overall ambiguity in the branching and low boot-strap values are due to the very high sequence identity within the *Ectothiorhodospira*, which extends not only to strains of the same species from different geographic areas, but also for entirely different species (e.g., *Ect. haloalkaliphila*, *Ect. magna*, *Ect. marina*, and *Ect. shaposhnikovii*). DNA-DNA hybridization results indicated that PHS-1 belongs to the same species as *Ectothiorhodospira shaposhnikovii* DSM 2111 (81.1% sequence similarity) and is a separate species from *Ectothiorhodospira shaposhnikovii* DSM 243^T^ (58.1% sequence similarity).

### 3.4. Analysis of the Anaerobic Arsenite Oxidase Gene arxA

**Figure 7:**
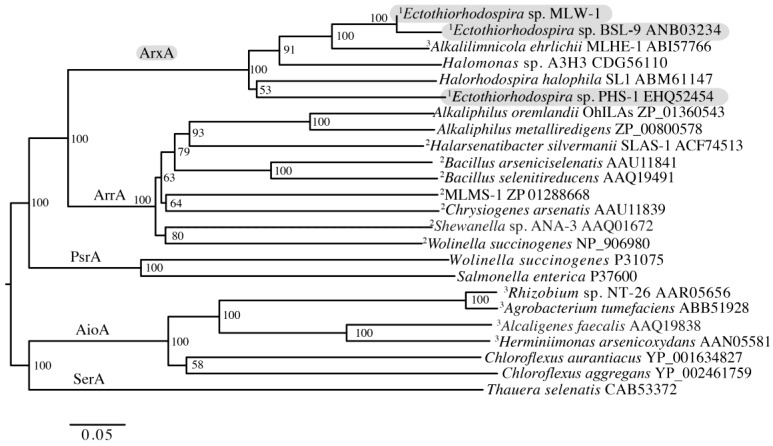
Phylogenetic analysis of ArxA-type arsenite oxidase, and other clades of DMSO reductase family molybdenum enzymes. The Genbank accession numbers are noted at the end of each OTU. KALIGN (http://www.ebi.ac.uk/Tools/msa/kalign/) was used for the multisequence alignment. The phylogenetic inference was done using PAUP using the distance criterion and neighbor joining for tree searching. Gaps within multi-sequence alignment were included, which resulted in 1099 possible positions for constructing the final tree. The tree was rooted on selenate reductase, SerA. Superscripts 1, 2, and 3 indicate that the organism oxidizes arsenite coupled to photosynthesis, respires arsenate, or oxidizes arsenite chemoautotrophically, respectively. Grey highlights the new species described in the current manuscript.

Molecular detection of genes encoding arsenite oxidases (*aioA* and *arxA*) by PCR indicated that PHS-1, MLW-1, and BSL-9 lacked *aioA* but contained *arxA* (Figure S3). *Halorhodospira halophila, Ectothiorhodospira shaposhnikovii* DSM 243^T^, and *Ectothiorhodospira shaposhnikovii* DSM 2111 contained *arxA* but lacked *aioA*. No *aio* genes were detected within the genome sequences for PHS-1 and BSL-9 [27]. For PHS-1 and BSL-9 the complete *arxA* genes were retrieved from their genome sequences and a partial *arxA* sequence for MLW-1 and for *Halorhodospira halophila* was determined from a PCR amplicon generated using primers from the BSL-9 *arxA* gene. Pairwise analysis among these *arxA* sequences indicated that PHS-1 *arxA* was the most divergent, with 70% and 73% sequence identity scores relative to BSL-9 and MLW-1, respectively. The *arxA* for the two strains was highly homologous as they share 98% DNA sequence identity (Figure 7). The lack of *aio* genes and the presence of *arx* gene clusters in PHS-1, MLW-1, and BSL-9 suggest that the mechanism for photosynthetic arsenite oxidation is likely associated with *arx* in these organisms.

## 4. Discussion

The overall goals of this work were to demonstrate As(III)-linked photosynthetic growth in new strains of *Ectothiorhodospira* taken from As-rich soda lakes, and to determine whether they all shared a similar genetic mechanism responsible for this oxidation (i.e., *arxA*). Clear evidence for robust As(III)-dependent anaerobic phototrophic growth was observed in strains MLW-1 and BSL-9 (Figure 1), as was previously reported for strain PHS-1 [4], as well as an *Ectothiorhodospira*-dominated enrichment culture established from Mono Lake water [3]. The three type culture strains were also able to oxidize As(III) (Figure 2A,C,E), but growth only occurred for *Ect. shaposhikovii* DSM 243^T^ and *Ect.* shaposhnikovii DSM 2111 in the presence of sulfide, with As(III) providing a marginal growth enhancement for the former but not the latter microorganism (Figure 2B,D). *H. halophila’s* growth was even more complicated to interpret in that we used a carbon source taken from the literature (succinate) as well as sulfide to achieve growth. When As(III) was provided as an electron donor in addition to sulfide and succinate, a modest stimulation of growth occurred (Figure 2F). Under reducing conditions, sulfide reacts with arsenite to form thioarsenates [31], thus it may be that these strains are growing on thioarsenates rather than As(III) or sulfide [32], but we did not pursue this question as it was beyond the scope of the investigation. The capacity to link electrons scavenged from As(III) oxidation to anoxygenic phototrophic growth appears to be a common feature amongst these haloalkaliphilic autotrophs. However, the ability to achieve this feat solely with As(III) as the electron donor seems to require the isolation of new strains that have been deliberately selected for this capacity as part and parcel of the cultivation process.

The currently described genetic pathways for arsenite oxidation include *aio* and *arx*. For the latter, it was first shown previously that As(III)-dependent growth in the chemoautotroph *Alkalilimnicola ehrlichii* required *arxA* [12]. For photosynthetic arsenite oxidation, genome sequencing analysis and gene expression observations of PHS-1 hinted at a possible *arx*-dependent pathway [14]. However, this was first confirmed in BSL-9 by demonstrating disruptive mutants of *arxA* in strain BSL-9 were unable to oxidize As(III) or achieve phototrophic growth with As(III) [33]. Closer inspection of the PHS-1 genome revealed an *arx* gene cluster (*arxB2ABC*) that closely aligned with the *arx* gene cluster of BSL-9 and MLHE-1 [14,33]. Moreover, MLHE-1, BSL-9, and PHS-1 lacked homologs to any of the currently known *aio* sequences. In light of these genome sequence observations and genetic studies with BSL-9 and MLHE-1, our PCR detection results suggest that PHS-1 and MLW-1 may also use the *arx* pathway for As(III) oxidation. Further genetic work is needed to test this hypothesis. For the other strains, our PCR results led to the detection of *arxA* in *H. halophila*, *Ect. shaposhnikovii DSM 243^T^*, and *Ect. shaposhnikovii DSM 2111* but not *aioA* genes (Figure S3), also suggestive of an *arx-*dependent pathway for arsenite oxidation, as was previously demonstrated for BSL-9 and MLHE-1. For *H. halophila*, its genome sequence also contains an *arx*-like gene cluster and its ArxA-like sequence falls within the ArxA clade (Figure 7). None of these three strains represent new species based on their 16S rRNA gene sequence similarities to the established *Ectothiorhodospira* strain RM1 (Figure 6) and, in the case of PHS-1, its close proximity to *Ect. shaposhnikovii DSM 2111* rather than *Ect. shaposhnikovii DSM 243^T^*, as established by DNA–DNA hybridization. Indeed, for the most part their growth-supporting substrate affinities for electron donors have far more compounds in common than not, including, in addition to arsenite, sulfide, thiosulfate, acetate, lactate, propionate, succinate, malate, and pyruvate (Table 1).

Physiological differences between the three strains are, with the exception of salinity, relatively minor and generally reflect the locales from which they were isolated. Strain PHS-1 came from a biofilm in a hot spring located on Paoha Island [4]. The spring water temperature was ~45 °C and, given a roughly 50–50 mix with perched freshwater, had a salinity and arsenic content about half that of Mono Lake water, while strain MLW-1 was taken from the shoreline of the lake and exposed to its full salinity. Hence, strain PHS-1 shows a slightly higher temperature preference (Figure 3A), a similar pH range (Figure 3B), but a markedly lower salinity tolerance (Figure 3C). All three strains were clearly alkaliphilic.

The *arx* genes have been elucidated relatively recently [11] when compared with the better studied *aio* genes that are commonly linked to aerobic As(III) oxidation [34]. Our work now broadens the occurrence of *arx* genes within photosynthetic bacteria isolated from three different environments located in two arsenic-rich soda lakes (Mono Lake and Big Soda Lake). They now include *Ectothiorhodospira* strain PHS-1, *Ectothiorhodospira* RM1 strain MLW-1, and *Ectothiorhodospira* RM1 strain BSL-9. The chemoautotroph *A. ehrlichii* has already been shown to possess the *arx* gene [12]. Hamamura et al. [15] reported the presence of *arxA* homologs in amplicons from sediment-extracted DNA from a Tukh Lake, a soda lake located in Mongolia. They also isolated a chemoorganotrophic nitrate-respiring bacterium, *Halomonas* strain ANAO-440, which possesses *arxA* and oxidized As(III) to As(V) during heterotrophic growth. A recent transcriptomic study documented in situ expression of the *arxA* gene in a photosynthetic biofilm on Paoha Island, Mono Lake [33], the site of earlier investigations of As(III)-linked anoxygenic photosynthesis [4,10]. A broad meta-transcriptomic study of the entire water column in Mono Lake found that expression of *arxA* genes was abundant at several depths, especially at and below the 15 m oxycline, while expression of *aioA* was confined to only one depth (10 m) in the oxic epilimnion. Moreover, transcripts of *arxA* were far more abundant overall than those of *aioA* [35,36]. Taken together, these two studies underscore the importance of expression of ArxA as a mechanism of As(III) oxidation in Mono Lake’s hot spring and water column environments. It remains to be seen whether *arxA* genes occur and are expressed in other types of As-rich environments (e.g., contaminated groundwater; acid mine drainages; neutral/acidic pH hot springs) besides soda lakes.

## Figures and Tables

**Table 1:** Phototrophic growth of *Ectothiorhodospira* strains on a diversity of electron donors. All organic/inorganic sources were at 5 mM unless otherwise noted. Growth indices (OD_680_) are as follows: +, growth; -, no growth; ND, not determined.

	MLW-1	PHS-1	BSL-9	*Ect. shaposhnikovii* (DSM 2111)	*Ect. shaposhnikovii* (DSM 243) Type Strain	*Halorhodospira halophila*
Arsenite (2 mM)	+	+	+	-	-	-
Sulfide	+	+	+	+	+	+ ^b^
Thiosulfate	+	+	+	+	+ ^a^	+ ^b^
Sulfur	+	+	+	+	+ ^a^	+ ^b^
Hydrogen	-	-	ND	-	+ ^a^	ND
Acetate	+	+	+	+	+	+ ^b^
Lactate	+	+	+	+	+	ND
Propionate	+	+	+	+	+ ^a^	ND
Fructose	+	-	ND	+	+ ^a^	ND
Glucose	+	-	ND	-	ND	ND
Succinate	+	+	+	+	+ ^a^	+ ^b^
Malate	+	+	+	+	+ ^a^	ND
Pyruvate	+	+	+	+	+ ^a^	ND
Butyrate	-	-	ND	+	-	ND

^a^ Imhoff [2]; ^b^ Raymond and Sistrom [21].

## Supplementary Materials

The following are available online at http://www.mdpi.com/2075-1729/7/1/1/s1.
