# Supplementary material for: Arsenite as an Electron Donor for Anoxygenic Photosynthesis: Description of Three Strains of Ectothiorhodospira from Mono Lake, California and Big Soda Lake, Nevada
Source: Life (Basel). 2016 Dec 26;7(1):1. doi: 10.3390/life7010001 (PMC5370401; doi:10.3390/life7010001)
Supplement: Supplementary file 1 [file life-07-00001-s001.pdf]

Supplementary Materials: Arsenite as an Electron Donor for Anoxygenic Photosynthesis: Description of Three Strains of *Ectothiorhodospira* from Mono Lake, California and Big Soda Lake, Nevada

Shelley Hoeft McCann, Alison Boren, Jaime Hernandez-Maldonado, Brendon Stoneburner, Chad W. Saltikov, John F. Stolz and Ronald S. Oremland

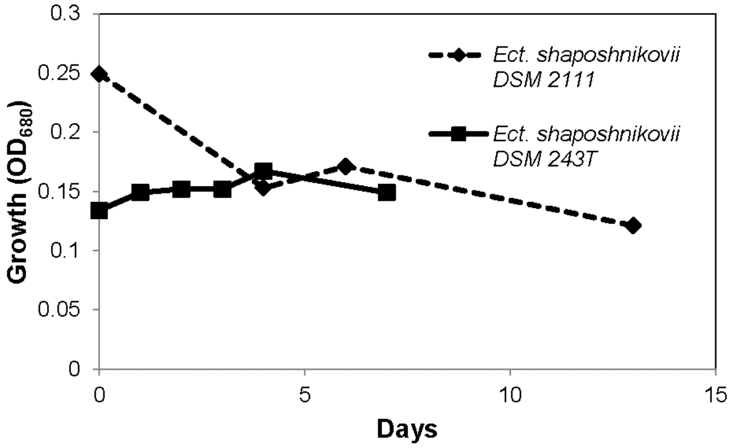

Figure S1. Growth data (OD_{680}, absorbance at 680 nm) of *Ect. shaposhnikovii* (DSM 2111) (and *Ect. shaposhnikovii* (DSM 243^T) when provided with 2 mM As(III) as the sole electron donor.

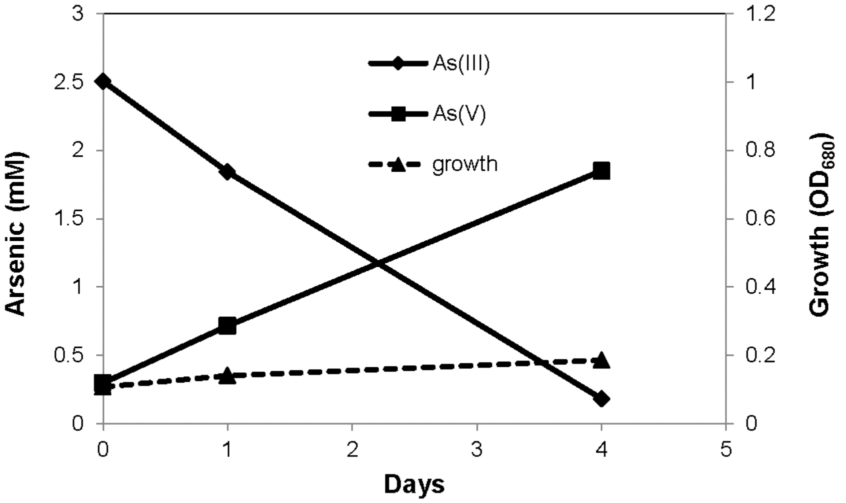

Figure S2. Anaerobic growth *of Halorhodospira halophila* grown phototrophically with As(III) as the sole electron donor.

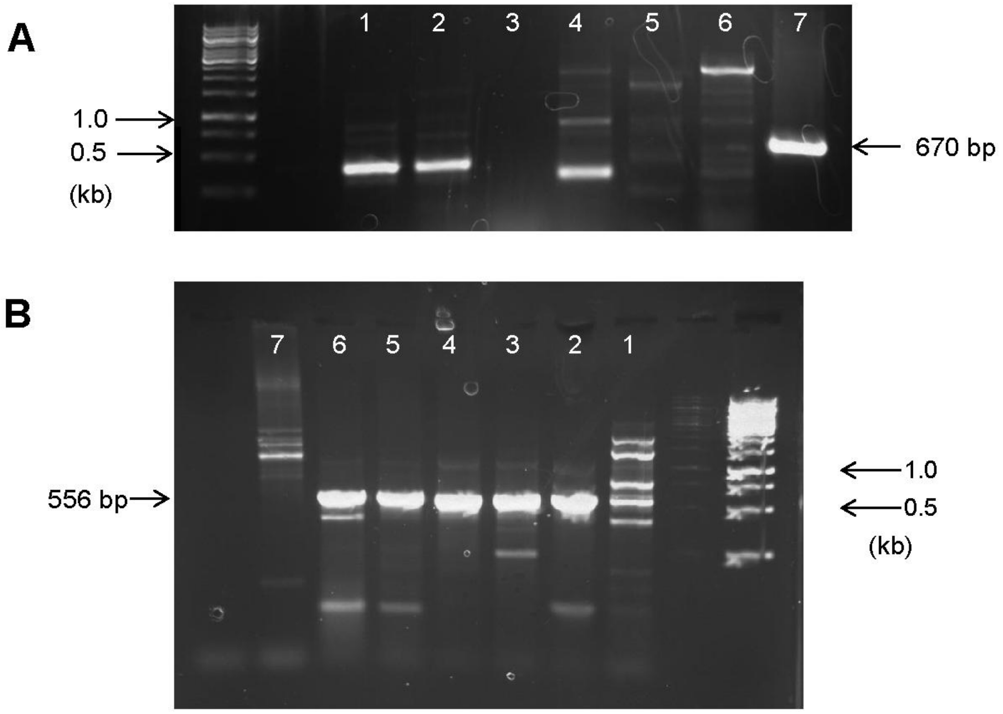

Figure S3. PCR analysis for (**A**) *aioA* (amplicon size = 670 bp) and (**B**) *arxA* (amplicon size 556 bp): (1) *Ect. shaposhnikovii* DSM 2111; (2) *Ect. shaposhnikovii* DSM 243^T; (3) *H. halophile*; (4) PHS-1; (5) MLW-1; (6) BSL-9; and (**7**) *Thermus thermophiles*.
